# Increased interleukin 1α and interleukin 1β expression is involved in the progression of periapical lesions in primary teeth

**DOI:** 10.1186/s12903-018-0586-3

**Published:** 2018-07-16

**Authors:** Ning-Yan Yang, Yan Zhou, Huan-Ying Zhao, Xiao-Yong Liu, Zheng Sun, Jia-Jian Shang

**Affiliations:** 1 Department of Pediatric Dentistry, Beijing Stomatological Hospital & School of Stomatology, Capital Medical University, Tian Tan Xi Li No. 4, Dong Cheng District, Beijing, China; 2 Medical Experiment and Test Center, Capital Medical University, Xi Tou Tiao No 10, You An Men Wai, Feng Tai District, Beijing, China; 3 Department of Oral Pathology, Beijing Stomatological Hospital & School of Stomatology, Capital Medical University, Tian Tan Xi Li No. 4, Dong Cheng District, Beijing, China; 4 Department of Oral Medicine, Beijing Stomatological Hospital & School of Stomatology, Capital Medical University, Tian Tan Xi Li No. 4, Dong Cheng District, Beijing, China

**Keywords:** Interleukin 1α, Interleukin 1β, Inflammation, Periapical periodontitis, Primary teeth

## Abstract

**Background:**

Interleukin 1 (IL-1) is involved in bone resorption. However, the role of IL-1 in periapical lesions characterized by periapical bone destruction in primary teeth has not yet been fully elucidated. This study aimed to detect the distribution and expression of IL-1 in periapical lesions in primary teeth and assess the relationship between the cytokines and the degree of inflammatory cell infiltration.

**Methods:**

A total of 106 chronic periapical lesions in primary teeth were harvested. Haematoxylin and eosin (H&E) staining was used to determine the histological type and the inflammatory cell infiltration grade (mild, moderate, and severe), and immunohistochemistry and enzyme-linked immunosorbent assay (ELISA) were used to detect the distribution and expression of IL-1α and IL-1β.

**Results:**

Of the 106 chronic periapical lesion samples, there were 85 cases of periapical granuloma, accounting for 80.19% of the total samples, and 21 cases of radicular cysts, accounting for 19.81%; no cases of abscess were detected. Immunohistochemistry results showed that both IL-1α and IL-1β were expressed in periapical granulomas and cysts. ELISA results showed that IL-1α and IL-1β levels were higher in the periapical granuloma group than in the radicular cyst and normal control groups (*P* < 0.05). In the periapical granuloma group, IL-1α and IL-1β were detected at higher levels in the severe inflammatory cell infiltration subgroup than in the mild-inflammatory cell infiltration subgroup (*P* < 0.05), and IL-1β expression was also higher in the moderate inflammatory cell infiltration subgroup than in the mild inflammatory cell infiltration subgroup (*P* < 0.01). A significant positive correlation was observed between the protein expression levels of IL-1α and IL-1β and the inflammation grade in periapical granulomas from primary teeth (*P* < 0.05).

**Conclusion:**

Expression levels of the cytokines IL-1α and IL-1β in periapical granulomas from primary teeth increased with increasing inflammatory severity and appeared to be a contributing factor to the progression of periapical lesions.

## Background

Chronic periapical periodontitis characterized by pathogenic bone resorption around the root apex is the major cause of premature primary tooth loss. In cases of severe infection, it can even lead to permanent hypoplastic teeth [1, 2], and this is very harmful to children. For a long time, bacterial infection in the dental pulp was considered to be the cause of periapical periodontitis [3]. However, humoural and cell-mediated immune reactions are also reported to be involved in the periradicular inflammatory processes in primary teeth [4]. In recent years, studies on chronic arthritis and periodontitis have suggested that multiple cytokines produced by both inflammatory and non-inflammatory host cells are involved in the mediation of bone resorption [5, 6].

The pro-inflammatory cytokine interleukin (IL)-1 is a key regulator of host responses to microbial infection and can enhance bone resorption and inhibit bone formation [7]. IL-1α and IL-1β are two members of the IL-1 family [8]. Martinez et al. reported that IL-1α and IL-1β accumulation was observed in periapical lesions by immunohistochemistry [9]. IL-1α is produced by a series of cell types, such as fibroblasts, osteoblasts, and neutrophils, in periapical lesions following pulpal infection [10]. IL-1β might also play a role in the initiation and up-regulation of the inflammatory response in apical periodontitis by increasing the levels of IL-6 and prostaglandin E2 production [11]. Research on persistent apical periodontitis by Morsani et al. suggested that increased IL-1β production may contribute to increased susceptibility to persistent apical periodontitis [12]. Although the effects of IL-1 have been extensively researched in permanent teeth, the effects remain unclear in apical periodontitis in primary teeth.

Therefore, the aim of this study is to investigate the distribution and expression of IL-1 in periapical lesions in primary teeth and assess the relationship between cytokines and the degree of inflammatory cell infiltration.

## Methods

### Patient selection

All clinical procedures performed in this study were approved by the Human Volunteers Research and Ethics Committee of Beijing Stomatological Hospital, Capital Medical University, Beijing, China ([2015]88). All procedures were explained in advance to the participating parents, and parental written informed consent was obtained for all patients. A total of 106 samples were collected from patients (5–9 yrs. of age, mean age 7.13 ± 1.76 yrs). Ten normal periodontal ligament samples from primary teeth were used as controls for sequence extraction. Primary molars diagnosed with chronic periapical periodontitis according to the criteria established by Torabinejad and Walton [13] without previous dental treatment and that had to be extracted were selected for this study. All extracted molars were at the stable root stage. No apparent communication between the periodontal pockets and the periapical lesions was detected by periodontal probe. Patients who presented with systemic diseases, teeth with sinus tracts or a periodontal probing depth more than 3 mm or who had received antibiotic therapy within 3 months prior to collection were excluded from the study.

### Sampling procedure

After local anaesthesia, the tooth and its surrounding area were isolated with cotton balls and swabbed with 1% iodine tincture. The periapical tissue was collected from both the socket and the furcation of the primary molar by sterile curette after extraction; the tissue was immediately washed with sterile saline to remove residual blood and planktonic bacteria. All periapical tissue samples were divided into two segments. One of them was embedded in paraffin wax and sent for histopathological examination. The other one was stored immediately in an Eppendorf tube with saline solution and frozen at − 80 °C for cytokine detection.

### Haematoxylin and eosin (H&E) staining and inflammation grade

Paraffin sections that were 4-μm-thick were heat immobilized, deparaffinized with xylene, and then rehydrated using a graded series of ethanol. H&E staining was performed, and histological evaluations were performed with a light microscope (Olympus Corporation, Tokyo, Japan). The histological types and inflammation grades of the chronic periapical periodontitis samples were determined by oral pathologists. The inflammation grades of the samples were evaluated as described previously [14]. Briefly, the H&E-stained sections were graded at 200× magnification based on the average inflammation in three consecutive fields. The grading criteria were as follows: mild, inflammatory cells composing less than 1/3 of the field; moderate, inflammatory cells composing 1/3 to 2/3 of the field; severe, inflammatory cells composing more than 2/3 of the field.

### Immunohistochemistry

Paraffin sections were used for immunohistochemistry. Briefly, the sections were first deparaffinized in xylene, rehydrated, washed with phosphate-buffered saline (pH 7.4) and incubated with 3% H_2_O_2_ for 15 min to block endogenous peroxidase at room temperature. The sections were then incubated with primary antibodies against IL-1α and IL-1β (1:100 dilution, Abcam, Cambridge, UK) in a humidified chamber at 4 °C overnight. After that, the sections were incubated with HRP-conjugated goat anti-rabbit secondary antibody at 37 °C for 30 min, treated with 3,3′-diaminobenzidine (DAB) for visualization, and then counterstained with haematoxylin.

### Enzyme-linked immunosorbent assay (ELISA)

IL-1α and IL-1β levels were measured by using ELISA kits (RD Biosciences, San Diego, CA, USA) according to manufacturer’s instructions. In brief, the periapical tissue samples were finely cut and homogenized in lysis buffer in ice. The tissue suspensions were pyrolysed by ultrasound and then centrifuged at 12000 g for 10 min at 4 °C; finally, the supernatants were collected. A total of 50 μl of serially diluted standard and samples were added to the ELISA plate wells and incubated with horseradish peroxidase-conjugated specific antibodies for IL-1α and IL-1β for 30 min at 37 °C. The cytokines values were detected at 450 nm and calculated with the standard curve. The total protein concentrations were assayed by the bicinchoninic acid method. The cytokine concentrations are expressed as μg/mg total protein.

### Statistical analysis

According to the data type, differences among the groups were analysed by the chi-square test, Student’s t test or Kruskal-Wallis H test. The relationships between the protein expression levels of IL-1α and IL-1β and the inflammation grade were analysed by Spearman’s rank correlation. All calculations were performed with SPSS for Windows, version 19.0 (SPSS Inc., Chicago, IL, USA). *P* < 0.05 was considered statistically significant.

## Results

### Characteristics of the clinical samples

We harvested chronic periapical periodontitis clinical samples from 106 patients, including 50 boys and 56 girls. Age, sex, and the different pathological types of chronic periapical lesions are summarized in Table 1. The mean age of the patients was not significantly different between the periapical granuloma and radicular cyst groups. There were no significant differences in sex between the two groups.

**Table 1:** Characteristics of the chronic periapical periodontitis samples

	n (%)	Age (X ± S)	Sex	
			Male	Female
Granuloma	85 (80.19%)	7.04 ± 1.81	39	46
Cyst	21(19.81%)	7.45 ± 1.61	11	10
Abscess	0	–	–	–

### Immunohistochemical localization of IL-1α and IL-1β in the periapical lesions from primary teeth

**Fig. 1:**
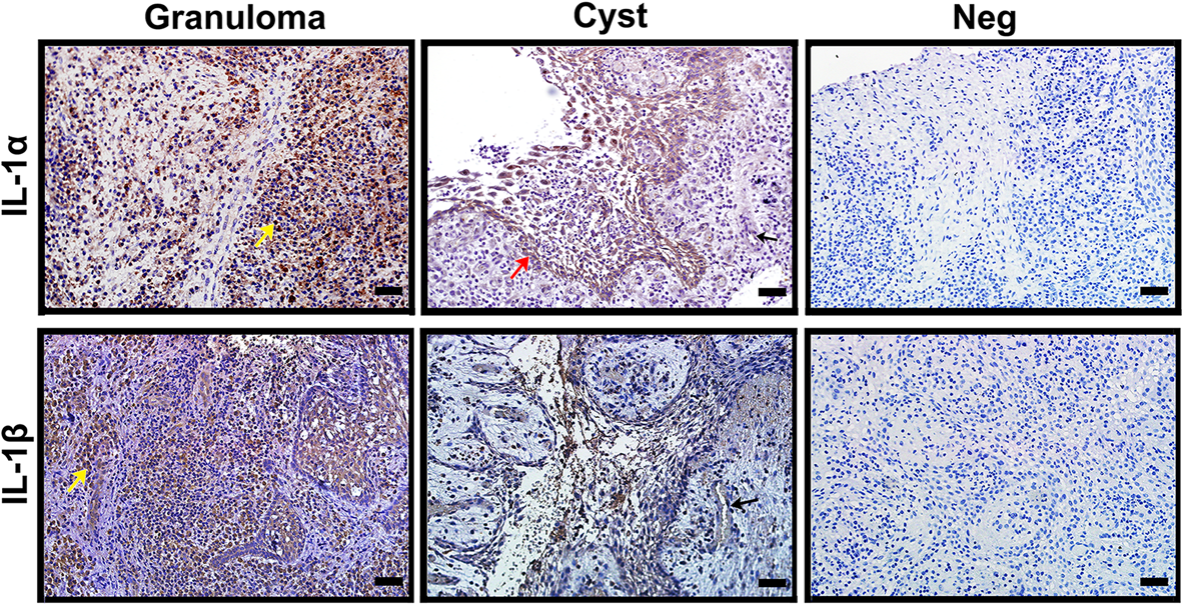
IL-1α and IL-1β distribution in periapical lesions. The IL-1-positive cells display brown staining. Neg: negative control. Yellow arrow: inflammatory cells. Red arrow: epithelial cells. Black arrow: vascular endothelium. The scale bar is 50 μm

In our study, we observed the IL-1α and IL-1β distribution in periapical lesions by immunohistochemistry. Our results showed that IL-1α and IL-1β were both expressed in periapical granulomas and radicular cysts (Fig. 1). In addition to inflammatory cells and epithelial cells, the cytokines were also expressed on the vascular endothelium.

### Quantitative IL-1α and IL-1β protein expression in periapical lesions from primary teeth

**Fig. 2:**
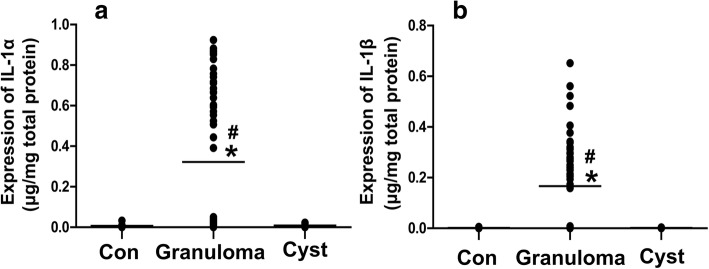
IL-1α (**a**) and IL-1β (**b**) expression levels in periapical granulomas and radicular cysts. Con, control (*n* = 10); Granuloma, periapical granuloma (*n* = 85); Cyst, radicular cyst (*n* = 21). * *P* < 0.05 compared with Con, # *P* < 0.05 compared with Cyst

ELISA was used to determine the IL-1α and IL-1β expression levels in periapical lesions. We found that the expression levels of IL-1α and IL-1β in the radicular cyst and normal control groups were very low; the average expression levels of IL-1β in those two groups were only 0.0017 and 0.0016 μg/mg total protein, respectively. In addition, both IL-1α and IL-1β expression levels were higher in the periapical granuloma group than in the radicular cyst and normal control groups (*P* < 0.05), whereas there was no significant difference between the radicular cyst and control groups (Fig. 2).

**Fig. 3:**
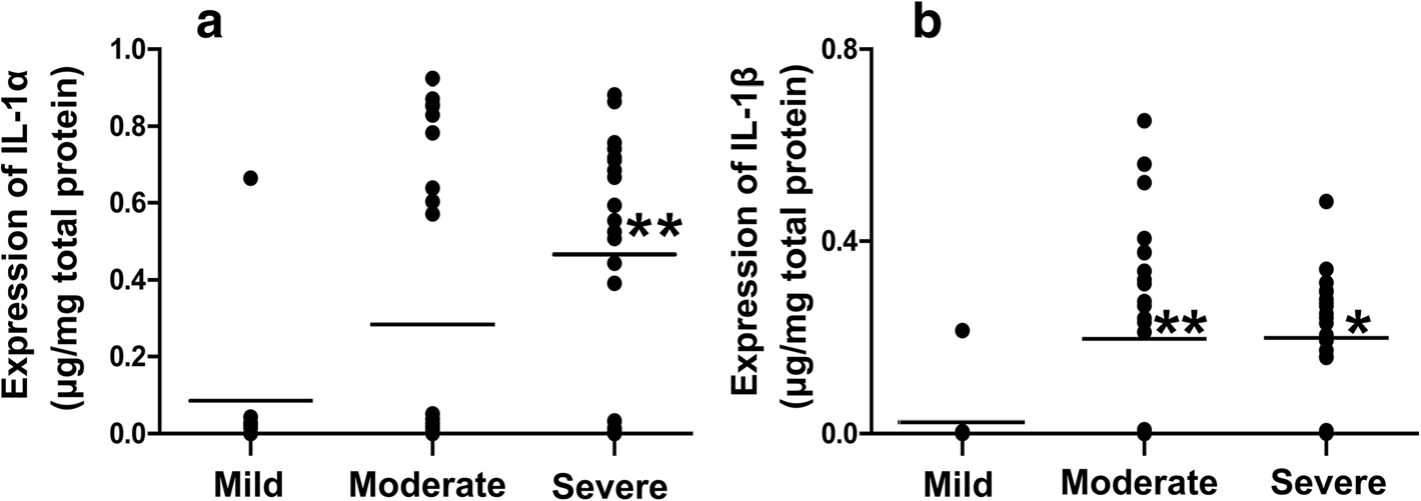
IL-1α (**a**) and IL-1β (**b**) expression levels at different inflammatory cell infiltration grades in periapical granulomas. Mild, mild inflammatory cell infiltration subgroup (*n* = 16); Moderate, moderate inflammatory cell infiltration subgroup (*n* = 32); Severe, severe inflammatory cell infiltration subgroup (*n* = 37). * *P* < 0.05 and ** *P* < 0.01 compared with the mild inflammatory cell infiltration subgroup

To further investigate the relationship between IL-1α and IL-1β expression and periapical granuloma progression, the periapical granuloma group was divided into three different inflammation grade subgroups, including 16 samples in the mild inflammatory cell infiltration subgroup, 32 samples in the moderate inflammatory cell infiltration subgroup, and 37 samples in the severe inflammatory cell infiltration subgroup. The results showed that IL-1α and IL-1β levels were higher in the severe inflammatory cell infiltration subgroup than in the mild inflammatory cell infiltration subgroup (*P* < 0.05 or *P* < 0.01, Fig. 3), and IL-1β expression was also higher in the moderate inflammatory cell infiltration subgroup than in the mild inflammatory cell infiltration subgroup (*P* < 0.01). IL-1α and IL-1β expression was, however, not significantly different between the severe and moderate inflammatory cell infiltration subgroups.

### Relationships between IL-1α and IL-1β protein expression levels and the inflammation grade in periapical granulomas from primary teeth

The relationships between the IL-1α and IL-1β protein expression levels and the inflammation grade in periapical granulomas from primary teeth were analysed by Spearman’s rank correlation (Table 2). We found that IL-1α and IL-1β expression levels were both associated with the inflammation grade in periapical granulomas (*P* < 0.05). Moreover, a significant positive correlation was observed between the protein expression levels of IL-1α and IL-1β in periapical granulomas from primary teeth (*P* < 0.01).

**Table 2:** Relationships between IL-1α and IL-1β expression and the inflammation grade in periapical granulomas from primary teeth

	IL-1α	IL-1β
Inflammation grade	0.301^a^*	0.320*
IL-1α	–	0.630**

^a^Spearman’s rank correlation coefficient (*P < 0.05, **P < 0.01)

## Discussion

In this study, we found that periapical granulomas are the major histopathologic type of periapical lesions in primary teeth. In addition, the cytokines IL-1α and IL-1β are abundantly expressed in periapical granulomas, and these high levels of IL-1α and IL-1β expression are consistent with the inflammation severity.

Notably, periapical lesions occur in fibrous and granulation tissue infiltrated by a large number of inflammatory cells, proliferating epithelium or cysts, or even abscesses [15]. Periapical granulomas, radicular cysts and periapical abscesses are three major histopathologic types of periapical lesions. Data from various studies have indicated a high incidence of periapical granulomas in permanent teeth, ranging from 50 to 84.2% [16–18]; however, Safi et al. reported a low incidence of 15.9% [19]. Furthermore, Mass et al. analysed 49 primary molars with radiolucent lesions and found that only 26.5% of the lesions were diagnosed as granulomas [20]. In our study, the incidence of periapical granulomas was 80.19%, which is in accordance with most of the previous studies. Moreover, the incidence of radicular cysts in the current study was 19.81%, which is in line with the findings of Saraf et al. and Sullivan et al. [18, 21]*.* In agreement with the low incidence of abscesses reported in the literature, there were no abscesses detected in our study. The lack of abscesses in our study might have been attributed to the sampling procedure, which failed to maintain the abscess intact [22] or the small sample size. Above all, the results suggested that the occurrence of periapical granulomas and radicular cysts in primary teeth was similar to that in permanent teeth.

It is well known that the destruction of periapical tissue is characterized by the development of granulomatous and cystic tissue, which then leads to bone resorption [23]. A number of studies have shown that IL-1 (including IL-1α and IL-1β) is closely associated with periodontal pathogenesis and the activation and recrudescence of osteoclastic bone resorption [10, 11, 24, 25]. Nair also stated that IL-1 plays a crucial role in stimulating lymphocytes, potentiating neutrophils, strengthening leukocyte adhesion, promoting bone resorption, and inhibiting bone formation [26]. However, most of the evidence linking periapical lesions to IL-1 comes from studies on permanent teeth. To better understand the role of IL-1 in periapical lesions in primary teeth, we investigated the expression of IL-1 in periapical lesions in primary teeth. Our immunohistochemistry results showed that positive IL-1α and IL-1β staining was found not only in the inflammatory cells but also in the epithelial cells and on the vascular endothelium in both periapical granulomas and radicular cysts from primary teeth. Our results were consistent with the findings from previous investigations showing that many cell types, such as mononuclear phagocytes, neutrophils and epithelial and endothelial cells, could all produce IL-1 and other cytokines [25]. Therefore, it has become clear that IL-1α and IL-1β are expressed in periapical lesions in primary teeth.

In addition, periapical granulomas and cysts represent different stages of the periapical inflammatory process. Periapical granulomas, the early stage of the infectious process, demonstrated a greater inflammatory response and more pathobiological activity [27]. A previous study also showed that higher expression of the cytokine RANKL was observed in periapical pathosis than in healthy pulpal tissue in deciduous teeth [28]. Similarly, our present observations indicated that higher expression levels of both IL-1α and IL-1β were found in the granuloma group compared to those in cyst and control groups. On the other hand, IL-1α and IL-1β levels in the cyst group were as low as those in the control group in the present study. As we know, periapical granuloma consists of granulomatous tissue, while radicular cysts usually have a central cavity that is lined by squamous epithelium and filled with fluid or semi-solid material [19]. In addition, it has been suggested that cyst fluid contains high concentrations of cytokines [29]. Thus, the loss of cyst fluid during the sampling procedure might be a possible explanation for the low concentrations of IL-1 detected in the cysts from primary teeth in our study. Taken together, the data suggest that IL-1α and IL-1β might be related to the inflammatory responses of periapical lesions.

To better understand the high expression levels of IL-1 in periapical granulomas, we analysed the changes in IL-1 expression at different inflammation grades of periapical granulomas. In our study, we found that IL-1α and IL-1β levels increased in the moderate and severe inflammatory cell infiltration subgroups of periapical granulomas. Particularly, our data from periapical granulomas from primary teeth showed a positive correlation between IL-1α and IL-1β expression levels and the inflammation grade, suggesting that increased IL-1α and IL-1β expression occurred parallel to the severity of the inflammation. Trebec-Reynolds et al. reported that both IL-1α and IL-1β activated osteoclasts, the cells responsible for bone resorption [30]. A number of studies have shown that higher levels of IL-1 were found in clinically symptomatic periapical lesions, which represent an immunologically active stage of periapical disease [15, 31]. Moreover, the increased levels of IL-1β and decreased level of IL-1 receptor antagonist (a naturally occurring inhibitor of the IL-1 receptor) in gingival crevicular fluid were related to the severity of adult periodontitis [32]. Another study on experimental rat periapical lesions indicated that IL-1β was more strongly expressed in the inflammation phase [9]. Based on the evidence, the results suggest a possible link between IL-1α and IL-1β production and the inflammation process in periapical lesions. It is well known that IL-1α and IL-1β are the two distinct ligands of IL-1, and those two ligands have high sequence homology and indistinguishable biological activities [33]. IL-1α and IL-1β are both essential for producing macrophages [34] and are associated with inflammatory disease [35]. Our data support that IL-1α in periapical granulomas from primary teeth was positively correlated with IL-1β. It seems that a synergic effect of IL-1α and IL-1β exists in the progression of periapical granulomas in primary teeth.

## Conclusions

In summary, the pro-inflammatory cytokines IL-1α and IL-1β are expressed in both periapical granulomas and radicular cysts in primary teeth. Furthermore, expression levels of the cytokines IL-1α and IL-1β in periapical granulomas in primary teeth increased with increasing inflammatory severity and appeared to be a contributing factor to periapical lesion progression. These findings might improve our understanding of the inflammatory development of periapical lesions.

## Abbreviations

DAB: 3,3′’-Diaminobenzidine
ELISA: Enzyme-linked immunosorbent assay
H&E: Haematoxylin and eosin
IL-1: Interleukin-1
